# Evaluating the feasibility of using the Multiphase Optimization Strategy framework to assess implementation strategies for digital mental health applications activations: a proof of concept study

**DOI:** 10.3389/fdgth.2025.1509415

**Published:** 2025-02-13

**Authors:** Ayla Aydin, Wouter van Ballegooijen, Ilja Cornelisz, Anne Etzelmueller

**Affiliations:** ^1^ Department of Clinical, Neuro and Developmental Psychology, Amsterdam Public Health Research Institute, Vrije Universiteit, Amsterdam, Netherlands; ^2^ Department of Educational and Family Studies, LEARN!, Amsterdam Center for Learning Analytics, Vrije Universiteit, Amsterdam, Netherlands; ^3^ HelloBetter, GET.ON Institut für Online Gesundheitstrainings GmbH, Hamburg, Germany; ^4^ School of Medicine and Health, Professorship Psychology & Digital Mental Health Care, Technical University of Munich, München, Germany

**Keywords:** DiGA, Digitale Gesundheitsanwendungen, digital health applications, implementation science, healthcare professionals, internet-based interventions, mental health, multiphase optimization strategy

## Abstract

**Background:**

Despite the effectiveness and potential of digital mental health interventions (DMHIs) in routine care, their uptake remains low. In Germany, digital mental health applications (DiGA), certified as low-risk medical devices, can be prescribed by healthcare professionals (HCPs) to support the treatment of mental health conditions. The objective of this proof-of-concept study was to evaluate the feasibility of using the Multiphase Optimization Strategy (MOST) framework when assessing implementation strategies.

**Methods:**

We tested the feasibility of the MOST by employing a 2^4^ exploratory retrospective factorial design on existing data. We assessed the impact of the implementation strategies (calls, online meetings, arranged and walk-in on-site meetings) individually and in combination, on the number of DiGA activations in a non-randomized design. Data from *N* = 24,817 HCPs were analyzed using non-parametric tests.

**Results:**

The results primarily demonstrated the feasibility of applying the MOST to a non-randomized setting. Furthermore, analyses indicated significant differences between the groups of HCPs receiving specific implementation strategies [*χ^2^* (15) = 1,665.2, *p* < .001, *ɛ*^2^ = 0.07]. Combinations of implementation strategies were associated with significantly more DiGA activations. For example, combinations of arranged and walk-in on-site meetings showed higher activation numbers (e.g., *Z* = 10.60, *p* < 0.001, *χ^2^* = 1,665.24) compared to those receiving other strategies. We found a moderate positive correlation between the number of strategies used and activation numbers (*r* = 0.30, *p* < 0.001).

**Discussion and limitations:**

These findings support the feasibility of using the MOST to evaluate implementation strategies in digital mental health care. It also gives an exploratory example on how to conduct factorial designs with information on implementation strategies. However, limitations such as non-random assignment, underpowered analysis, and varying approaches to HCPs affect the robustness and generalizability of the results. Despite these limitations, the results demonstrate that the MOST is a viable method for assessing implementation strategies, highlighting the importance of planning and optimizing strategies before their implementation. By addressing these limitations, healthcare providers and policymakers can enhance the adoption of digital health innovations, ultimately improving access to mental health care for a broader population.

## Introduction

Despite the proven effectiveness of various treatment options for mental health conditions [e.g., (1–3)], a considerable number of individuals with mental health conditions remain without appropriate care, resulting in a significant treatment gap (4, 5). This highlights the significant ongoing burden that mental health conditions pose on global health (6). Digital mental health interventions (DMHIs) can bridge the treatment gap by increasing access to support and resources (7), overcoming barriers like cost, time, and attitudinal preferences for self-management (8). However, the uptake of digital mental health interventions by healthcare professionals (HCPs) and patients outside of research studies is often low (9).

To increase patient access to digital health interventions, the German parliament passed the Digital Care Act (DVG) in 2019. This law established specific mobile and web applications [Digitale Gesundheitsanwendungen (DiGA)] as an integral part of the German healthcare system (10). DiGA are CE-marked (Conformité Européenne) medical devices of low-risk classes that can be prescribed to support insured persons in the treatment of illnesses. The CE certificate indicates that products sold within the European Economic Area (EEA) have been evaluated to ensure compliance with high safety, health, and environmental protection standards (11). DiGA must pass safety, functionality, data protection, and security tests at the Federal Institute for Drugs and Medical Devices [Bundesinstitut für Arzneimittel und Medizinprodukte (BfArM)] and show a medical benefit and patient-relevant structural and procedural improvements (12). DiGA cover a wide range of treatments, including mental health treatments and can be prescribed by HCPs (13). For example, the DIGA *HelloBetter Stress and Burnout* is a digital program to manage stress-related symptoms (14).

Despite the effectiveness of DMHIs as demonstrated by numerous randomized controlled trials (RCTs, e.g. (15–18), and the integration of DiGA into the German healthcare system, DiGA are not yet widely adopted. The report from Techniker Krankenkasse shows that by June 2023, 12% of physicians (22,200 out of 185,000) had prescribed DiGA. General practitioners accounted for 38% of prescriptions, while psychotherapists and psychiatrists made up 15%. Additionally, 74% of prescribers issued a maximum of two DiGA prescriptions (19).

Several barriers contribute to this limited uptake. From a patient perspective, concerns regarding data security and privacy present significant obstacles, particularly for vulnerable demographics such as older adults or those with limited digital literacy (7). From a HCPs perspective, HCPs often lack both knowledge and experience when it comes to the use of DiGA for the treatment of mental disorders. A survey conducted in 2023 revealed that 65% of HCPs were unaware of the specific benefits of DiGA, and 45% reported difficulty navigating the technical processes required for prescribing these tools (20). Providing information on the benefits of DiGA and training in new technologies can enhance awareness and knowledge (20). This is of significant importance, as HCPs are often the first touchpoint for patients seeking information about digital treatment options due to their role as gatekeepers in the healthcare system (21). System-level barriers also play a critical role. DiGA are rarely embedded in routine care pathways and the existing benefits are often communicated as unclear at the outset (22).

Addressing these barriers is essential to improve the uptake of DiGA. Implementation science offers a structured approach to address factors hindering uptake of an innovation such as DiGA. Here, “implementation strategies” are defined as a range of approaches or techniques that can be employed to enhance the adoption, implementation, sustainability and scale-up of innovations (23). In the field of healthcare, implementation strategies frequently entail educational meetings, use of local opinion leaders, patient mediated interventions and a combination of multi-faceted strategies (24).

Given that healthcare systems have limited resources for active implementation, implementation science may assist in addressing this issue effectively. Implementation science uses frameworks to structure, guide, analyze, and evaluate implementation efforts (25). Implementation science provides a wide range of theories, frameworks and methods to evaluate implementation processes. For example, the Consolidated Framework for Implementation Research (CFIR) aims to predict or explain barriers and facilitators to foster implementation effectiveness and success (26). By applying this framework, researchers might analyze contexts to tailor fitting implementation strategies to or evaluate factors fostering implementation processes. Another well-known example of an implementation framework is RE-AIM. This framework evaluates health interventions across five dimensions: Reach (individuals affected), Effectiveness (impact), Adoption (setting-level uptake), Implementation (fidelity and cost), and Maintenance (sustainability). The RE-AIM framework focuses on both individual- and setting-level outcomes, making it a valuable tool for evaluating intervention success comprehensively (27). While allowing for a comprehensive evaluation of the implementation process, the RE-AIM framework mainly focuses on the effect of the intervention and its context.

Developed as a guide for intervention developers in the fields of engineering, statistics, biostatistics, and behavioral science, the Multiphase Optimization Strategy (MOST) framework (28) depicts an innovative approach in analyzing intervention effects. MOST is a framework that integrates various perspectives from engineering, statistics, biostatistics, and behavioral science to optimize interventions and evaluate them in RCTs. The approach comprises three phases: preparation, optimization, and evaluation. It is employed for the purpose of optimizing and evaluating intervention components and their combinations to achieve the best possible outcomes. It emphasizes efficiency, effectiveness and resource allocation by means of a systematic testing and refining of intervention components across multiple phases (28).

Commonly, effectiveness evaluations of implementation strategies target singular implementation strategies or are confronted with interactions of multi-faceted implementation confounding results on actual effects. Defining *implementation strategies* as an *intervention* done to increase the uptake of certain practices, such as DiGA, researchers might be able to use the MOST framework to investigate the effect of implementation strategies quantitatively. This process might be of interest as well-known implementation frameworks do not allow for a quantitative and direct comparison of the effects of specific implementation strategies and their combinations. In contrast to implementation frameworks such as CFIR or RE-AIM, MOST provides a structured approach for systematically optimizing intervention components. The MOST framework puts a distinctive emphasis on the systematic optimization of intervention components (here: implementation strategies) using quantitative methods, making it well-suited for the evaluation of interactions and combined effects of multiple strategies in complex implementation contexts, such as digital health. Furthermore, in comparison with the CFIR and RE-AIM frameworks, MOST enables direct measure of the effect of specific implementation strategies and their combinations, potentially maximizing effectiveness of implementation strategies. Furthermore, MOST places particular emphasis on the systematic optimization of intervention components. Using this feature, researchers might be able to systematically optimize implementation strategies and processes.

To investigate the feasibility of utilizing the MOST framework to analyze implementation strategies in real-world settings, this proof-of-concept study was conceptualized. A proof-of-concept study aims to determine the workability of an application. Unlike full-scale studies, a proof-of-concept study is not designed to provide comprehensive results or solutions. Instead, its purpose is to demonstrate that the core idea has potential and is worth further investigation. Based on the findings of a proof-of-concept study, decisions can be made on whether to invest additional resources in further developing the concept (29).

For this study, we decided to focus on the first two phases of the MOST framework to align with the study's proof-of-concept nature. For this purpose, the preparation phase will focus on identifying and selecting potential implementation strategies. The optimization phase will systematically test combinations of identified implementation strategies to determine if it is feasible to use MOST in investigating the effect of implementation strategies in increasing DiGA activation numbers.

MOST's evaluation phase, which typically involves conducting a RCT to confirm the efficacy of the optimized intervention, will not be included in this study as the study's primary objective is to explore the feasibility of using the MOST framework to assess how effective implementation strategies can be for increasing DiGA activations. Conducting an RCT at this stage will be beyond the scope of this exploratory research and would require additional resources, planning and data collection.

Given the high potential but low uptake of DiGA as of yet it is important to explore how implementation strategies could be used to enhance DiGA uptake. Therefore, the objective of this proof-of-concept study is to demonstrate the feasibility of employing the MOST framework for assessing effects of implementation strategies for increasing DiGA activations.

An improved understanding of whether the MOST framework can be used to investigate the impact of implementation strategies on the number of DiGA activations will assist in the optimization of these strategies. This in turn could enable DiGA providers to optimize their efforts to engage with HCPs and improve their digital health interventions, ultimately leading to an improved uptake of DiGA.

## Methods

This proof-of-concept study aimed to assess the feasibility of using the MOST framework (28) to evaluate effects of implementation strategies for increasing DiGA activations. To demonstrate the feasibility of the MOST framework, we apply its first two parts using the preparation and optimization phases to investigate if the framework might be able to assess the effects of implementation strategies aimed at increasing DiGA activations. In the preparation phase, researchers identify and select intervention components (here: different implementation strategies) based on theoretical and empirical considerations. In the Optimization phase researchers test various configurations of these components to determine the most effective combination.

### Research design

This study is a proof-of-concept designed to demonstrate the application of the first two phases of the MOST framework to evaluate if the model can be used to assess the effects of implementation strategies for increasing DiGA activations through an exploratory retrospective factorial design.

In this study, we will consider the implementation of DIGA as the “intervention” and “implementation strategies” as “intervention components” in the MOST framework. DiGA activation numbers served as the intervention outcome of interest. Hereafter, the term “activation numbers” will be used to refer to the uptake or number of DiGA users, as the actual use of the DiGA is defined by the activation of the prescription.

In the preparation phase of the MOST framework, we developed a logic model as a structured framework for activities, outputs, and outcomes to identify and select implementation strategies. In the optimization phase, we employed an exploratory retrospective factorial design to systematically test different combinations of implementation strategies (28).

#### The logic model

**Figure 1:**
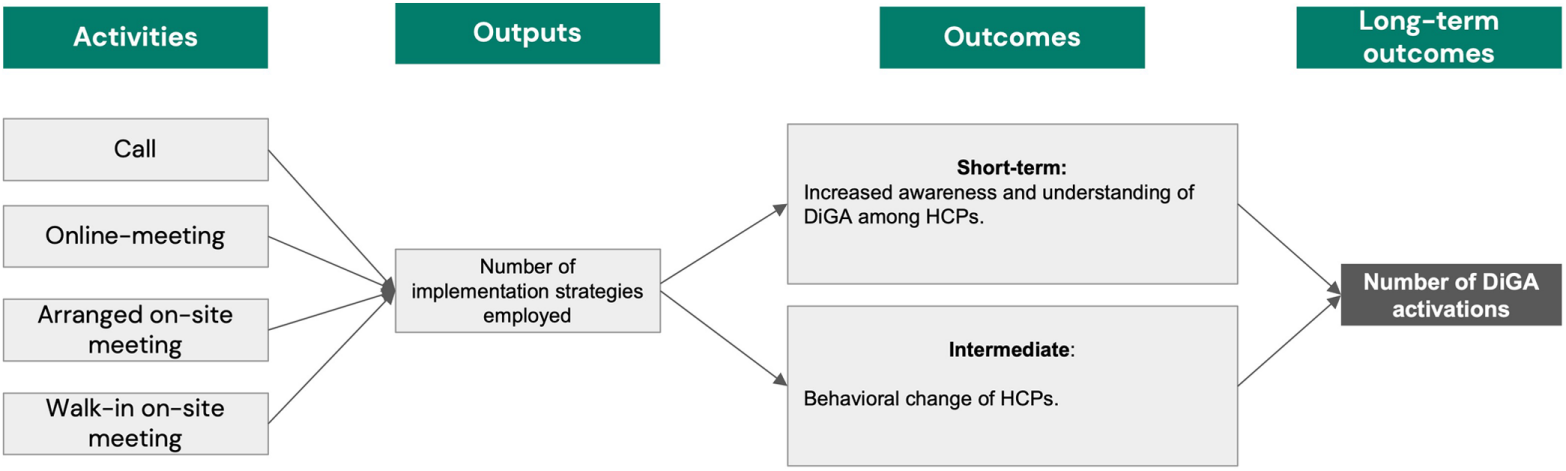
Logic model developed during the preparation phase of the MOST framework. The figure illustrates the logic model developed during the preparation phase of the MOST framework. Activities represent the implementation strategies employed (e.g., calls, online meetings, arranged on-site meetings, walk-in on-site meetings). Outputs refer to the number of strategies applied. Outcomes are categorized as short-term (increased awareness and understanding of DiGA among HCPs) and intermediate (behavioral change of HCPs). The long-term outcome is the number of DiGA activations.

Logic models are graphic tools that support design, planning and evaluation by visually organizing information and clarifying complex relationships. They describe planned actions and their expected results (30). The logic model of this study (Figure 1) depicts the activities that were undertaken to improve the DiGA activation numbers. These activities included implementation strategies such as calls, online-meetings, arranged and walk-in on-site-meetings. The implementation strategies were applied by HelloBetter employees. All calls and meetings were tailored to the HCP's specific interests. The focus of the calls and meeting was first, to introduce the HelloBetter DiGA and explain the prescription process of DiGA in general. Subsequently, the emphasis in the calls and meetings shifted to patient feedback, updates from HelloBetter, and the option of therapy progress reports. Generally, the objective was to provide comprehensive information to the HCPs and to inform about the benefits of HelloBetter DiGA.

The outcome described in the logic model was the number of distinct implementation strategies carried out. In the short term, we expected these strategies to increase HCPs awareness and understanding of DiGA. In the intermediate term, we anticipated that the strategies would change attitudes, subjective norms, and perceived behavioral control among HCPs, ultimately leading to more DiGA prescriptions. In the long term, we estimated to achieve an increased number of activations of DiGA.

#### The factorial design

In the optimization phase of our study, we employed a 2^4^ exploratory retrospective factorial design, exploring four factors (e.g., calls, online-meetings, arranged and walk-in on-site-meetings). Each strategy was either present (implemented) or absent (not implemented) for one individual HCP (data point), resulting in 16 unique experimental conditions (Table 1). These conditions allowed for the systematic examination of both the individual and combined associations of the strategies with the primary outcome. For example, one HCP received only phone calls and arranged on-site meetings, while another HCP received a combination of all four strategies.

**Table 1:** Number of HCPs in groups of the factorial design and descriptive statistics.

Grp.	Call	Online meeting	Arranged on-site-meeting	Walk-in on-site-meeting	*N* = 24,817	IQR	Median	Mean rank
1	No	No	No	No	21,298	1.00	0.0	11,838.81
2	Yes	No	No	No	671	2.00	0.0	14,930.64
3	No	Yes	No	No	121	1.00	0.0	12,915.57
4	Yes	Yes	No	No	67	1.50	0.0	14,265.66
5	No	No	Yes	No	1,078	2.00	0.0	15,128.76
6	Yes	No	Yes	No	102	2.75	0.5	15,514.10
7	No	Yes	Yes	No	20	1.50	1.0	15,832.62
8	Yes	Yes	Yes	No	29	4.00	1.0	16,372.45
9	No	No	No	Yes	1,012	2.00	1.0	16,369.88
10	Yes	No	No	Yes	34	1.00	1.0	18,712.10
11	no	Yes	No	Yes	12	6.25	1.5	16,402.33
12	Yes	Yes	No	Yes	10	2.25	3.0	22,238.60
13	No	No	Yes	Yes	319	4.00	2.0	19,122.94
14	Yes	No	Yes	Yes	29	7.00	2.0	19,865.88
15	No	Yes	Yes	Yes	7	9.00	2.0	17,410.36
16	Yes	Yes	Yes	Yes	8	7.25	3.5	21,426.38

Grp, group; IQR, interquartile range.

Each factor in a factorial design has its own set of controls and subjects (here: HCPs) can simultaneously serve as controls for some factors and treatments for others (28). The outcome of this research was the number of prescribed DiGA, operationalized by the DiGA activations registered on the therapy platform.

The retrospective nature of the design involved analyzing pre-existing data from HelloBetter's implementation efforts, where these strategies were applied in various combinations across HCPs. HCPs were not randomized into a specific condition. Instead, implementation strategies were applied randomly to the individual HCP. The retrospective approach allowed the study to utilize real-world data to explore the feasibility of applying the MOST framework without requiring new data collection.

### Setting

The data analyzed in this study was provided by HelloBetter, one of Germany's leading providers of DiGA. HelloBetter's digital therapy programs are available either online or via a mobile application and cover a wide variety of digital mental health interventions for the treatment and prevention of common conditions, such as stress and burnout, panic disorder or chronic pain.

HelloBetter collects data routinely while collaborating with HCPs working in various healthcare institutions, including clinics and private practices across Germany. Furthermore, the company documents their implementation strategies when engaging with HCPs. These routinely collected data were used for this study.

### Participants

HCPs work in organizations such as private practices or clinics across Germany and could potentially prescribe a DiGA. For this study, we focused on information on the group of HCPs who are medical doctors or psychotherapists and have recently been in contact with HelloBetter or prescribed a HelloBetter DiGA in the past. The study involved HCPs from a variety of practice settings across Germany. While the specific characteristics of these settings (e.g., urban or rural, practice size) were not the primary focus of this study, the application of the MOST framework allows for future investigations to explore how contextual factors influence the effectiveness of implementation strategies.

### Sample size

A *post-hoc* power analysis was conducted to determine the sample size required for a 2^4^ factorial design, which includes 16 experimental groups. The analysis considered an expected small effect size (Cohen's *f* = 0.10), as suggested by the literature on implementation strategies (31). An alpha level of 0.05 and a power of 0.80 were used as parameters for the analysis. The power analysis indicated a required total sample size of *N* = 1,904, with a sample size of *n* = 119 per group, to ensure sufficient power.

### Procedures

We analyzed existing data from January 14, 2022 to June 19, 2024, routinely collected by HelloBetter from several healthcare institutions across Germany. Participants regularly share information on their prescriber with HelloBetter to help with the communication in the prescription process. This information was used in this study to conduct a combined analysis on implementation strategies. Implementation strategies and activation numbers are routinely documented by HelloBetter and used for analysis in this study.

### Implementation intervention

Following the framework recommended by Proctor et al. (32), the implementation strategies utilized in this study were designed to enhance the adoption and integration of DiGA by HCPs. These strategies included phone calls, online meetings, and both arranged and walk-in on-site meetings. All strategies were implemented by trained HelloBetter employees and tailored to meet the preferences and specific needs of HCPs. The tailoring of strategies aligns with Proctor's emphasis on adapting implementation approaches to the target audience to maximize effectiveness.

Phone calls provided information about DiGA and HelloBetter programs, offered opportunities for HCPs to ask questions, and included the option to schedule follow-up online or on-site meetings. Online meetings covered the same content but also included product demonstrations for enhanced engagement and understanding, aligning with Proctor's focus on training and technical assistance. On-site meetings, whether arranged or spontaneous, allowed for face-to-face interaction in HCP practice settings and provided the added advantage of leaving physical informational materials directly at the practice. All interactions began with employees inquiring about the HCP's individual needs, ensuring that the content was relevant and practical, as Proctor's framework emphasizes personalization and responsiveness to stakeholder priorities.

A notable distinction among these strategies was the level of planning required. Online and arranged on-site meetings were always pre-scheduled, allowing for detailed preparation, while walk-in meetings were spontaneous, enabling dynamic engagement. Phone calls offered flexibility, being either pre-scheduled or spontaneous based on the situation. This multifaceted approach reflects Proctor's recommendation to balance structured and adaptable strategies, ensuring effective and context-specific implementation efforts.

HelloBetter employees incorporated detailed information about DiGA to inform and engage HCPs during the implementation process. Specifically, this included an overview of DiGA programs offered by HelloBetter, their intended use cases (e.g., treatment of mental health conditions such as stress and anxiety), and their clinical benefits as demonstrated in RCTs [e.g., (33–35)]. Additional details included eligibility criteria for patients, the process for prescribing DiGA, and practical considerations such as data privacy and reimbursement. This information was integrated into the various implementation strategies to ensure that HCPs could fully understand and adopt these tools.

### Variables and measures

The data used in this study included HCP gender, profession, discipline as well as the activation numbers of HelloBetter DiGA. Furthermore, HelloBetter documents various implementation strategies, including in-person implementation strategies such as calls, online-meetings, arranged and walk-in on-site-meetings.

### Outcomes

The primary aim of this study was to evaluate the feasibility of using the MOST framework to assess implementation strategies for increasing the number of DiGA activations. The secondary aim focused on examining the association between different implementation strategies—such as calls, online meetings, arranged, and walk-in on-site meetings—and the number of DiGA activations using the MOST framework. The number of DiGA activations was linked to HCPs having received different implementation strategies. Additionally, we sought to evaluate the number of DiGA activations in relation to the use of a combination of multiple implementation strategies and to explore the number of DiGA activations in relation to the total number of implementation strategies employed.

### Data analysis

We applied a retrospective factorial design representing the second step of the procedure of the MOST framework (28). The assumption of homoscedasticity was not met in our data, as indicated by Levene's test (*p* < .001), and the assumption of normality was not met, as indicated by the Anderson–Darling normality test (*p* < .001) on both the raw and Box-Cox transformed data. Therefore, the initially planned factorial ANOVA could not be carried out.

In light of the aforementioned violation of the assumptions, we applied the non-parametric Kruskal–Wallis rank test, which is more robust to different group sizes. A *post-hoc* analysis, namely Dunn's test with Bonferroni correction as recommended by (36), was performed to identify which specific groups differed from one another. In the Dunn's test, the order of the implementation strategies received by the groups is as follows: calls, online meetings, arranged on-site meetings, and walk-in on-site meetings. A “0” indicates that the specific implementation strategy was not received. The z-score in the Dunn's test indicates the magnitude and direction of the differences between groups. A positive z-score indicates that the first group in the comparison has higher activation numbers than the second group. Conversely, a negative z-score indicates that the first group in the comparison has lower activation numbers than the second group. Mean ranks were calculated to gain insight into which groups exhibited higher or lower values relative to the other groups.

### Pre-registration

In accordance with open science practices, this study was pre-registered on 12 June 2024 on the Open Science Framework (OSF) and can be accessed via https://osf.io/ugm2x.

### Ethical considerations

The study does not analyze any personal or sensitive data. The data available for this study was fully anonymized.

## Results

### Primary outcomes

#### The feasibility of applying the MOST framework to assess effects of implementation strategies

Within this study, the application of the first two phases of MOST framework proved feasible within the constraints of existing data, demonstrating its adaptability to non-randomized settings and varying group sizes.

Results indicate the feasibility of applying the MOST framework to evaluate effects of implementation strategies for increasing DiGA activations in real-world settings. By employing a retrospective factorial design, we systematically analyzed individual and combined implementation strategies, revealing effects of singular implementation activities and their combinations. Detailed results allow for the comparison of effects of implementation strategies. Using a factorial design, we were able to assess correlations between strategies and the number of DiGA activations. By conceptualizing DiGA implementation as an “intervention” and the various implementation strategies as “intervention components,” the study effectively tested the applicability of the MOST framework in this context.

### Secondary outcomes

#### Logic model

The logic model (Figure 1) developed during the preparation phase provided a structured framework for systematically identifying four strategies: calls, online meetings, arranged and walk-in on-site meetings. While specific outcomes directly related to the short-term and intermediate outcomes were beyond the scope of this study, it served as a tool to align the implementation strategies with the study's long-term outcomes of increasing DiGA activation numbers.

#### Descriptive statistics

Data on a total number of *N* = 24,817 HCPs, medical doctors and psychotherapists, were included in the analysis. As mentioned above, the power analysis required a total sample size of *N* = 1,904, with a sample size of *n* = 119 per group, to ensure sufficient power. However, due to limitations of working with existing data, the final group sample sizes did not meet the required sample sizes as the HCPs were not equally distributed across groups. Table 1 presents the sample sizes for the specific groups in the factorial design. As the required sample sizes were not met, the design was underpowered.

The interquartile range (IQR) values for the number of activations exhibited considerable variation across different groups (Table 1). For example, Group 15 has the largest IQR of 9.00, indicating substantial variability in the middle 50% of its data, while Group 10 has the smallest IQR of 1.00, indicating less variability.

Median values also varied, with groups like Group 5 (arranged on-site meeting) showing a median of 0.0, indicating no activations for at least half of the participants. Conversely, groups like Group 13, Group 14, and Group 15 had medians of 2.0, indicating that at least half of the participants had two or more activations (Table 1).

Mean ranks were calculated to gain insight into which groups exhibited higher or lower values relative to the other groups (Table 1). For instance, Group 3 (online meetings) had a low mean rank of 12,915.57, indicating lower activations compared to other groups. Group 5 (arranged on-site meetings) and Group 7 (online meetings and arranged on-site meetings) had moderate mean ranks, reflecting moderate activations. Group 12 (calls, online meetings, and walk-in on-site meetings) had the highest mean rank of 22,238.60, indicating the highest activations among the groups. The mean ranks of all groups can be found in Table 1.

#### Sample characteristics

The sample displayed a gender distribution with 57.97% female HCPs (*n* = 14,269). The sample consisted primarily of medical doctors with 84.43% (*n* = 20,954), while psychotherapists made up 15.57% of the sample. The disciplines were diverse, with the most prevalent being general medicine (23.83%), psychiatry and psychotherapy (17.06%), neurology, psychiatry, and psychotherapy (12.13%), and internal medicine (9.68%). Further descriptive information can be found in Table 2.

**Table 2:** Gender, profession, and discipline of healthcare professionals.

	*N* = 24,817
Gender	
Female	14,269
Male	10,363
Divers	5
Not specified	180
Profession	
Medical doctor	20,954
Psychotherapist	3,863
Discipline	
Anaesthesiology	371
Endocrinology and diabetology	204
General medicine	5,912
Gynaecology and obstetrics	1,243
Internal medicine	2,402
Neurology, psychiatry and psychotherapy	3,008
Occupational medicine	46
Orthopedics and trauma surgery	165
Otorhinolaryngology	103
Pediatrics and adolescent medicine	90
Physical and rehabilitative medicine	43
Psychiatry and psychotherapy	4,231
Psychosomatic medicine and psychotherapy	390
Sleep medicine	49
Surgery	97
Urology	32
Other	366
NA	6,065

NA = Not applicable: Healthcare professionals did not state their discipline.

#### Group differences

The results indicated significant differences between the groups (e.g., No Intervention, Online only, Call only, Walk-in only, Call + Online, Call + Walk-in, Online + Walk-in, etc.), *χ^2^* (15) = 1,665.2, *p* < .001. An epsilon-squared (*ε*^2^) value of 0.07 suggests a medium effect size.

Given that the Kruskal–Wallis test was statistically significant, *post hoc* pairwise comparisons using Dunn's test with Bonferroni correction revealed several statistically significant differences between the activation numbers of various groups. Table 3 illustrates the results of pairwise comparisons between experimental groups, highlighting *Z*-scores and adjusted *p*-values for each comparison. A comparison of groups that received any strategies with those that received no strategies revealed that the former exhibited consistently higher numbers of activations. For instance, comparisons involving the “No Intervention” group (0.0.0.0) consistently show large *Z*-scores and highly significant *p*-values when contrasted with groups receiving individual strategies or combined approaches. Comparison 1 in Table 3 demonstrates that the first group in the comparison (No Intervention) has a lower mean rank (fewer activations) than the second group (Walk-In Only) (*Z-*value = −24.22, *p* < .001, *χ^2^* = 1,665.24).

**Table 3:** Pairwise comparisons (Bonferroni-adjusted *P*-values) with *Z*-values of implementation strategies.

Nr of comparison	Comparison	*z*	*p*	*p*-adjusted	Significance
1	0.0.0.0–0.0.0.1	−24.220	0.000	0.000	*
2	0.0.0.0–0.0.1.0	−18.124	0.000	0.000	*
3	0.0.0.1–0.0.1.0	4.877	0.000	0.000	*
4	0.0.0.0–0.0.1.1	−22.208	0.000	0.000	*
5	0.0.0.1–0.0.1.1	−7.374	0.000	0.000	*
6	0.0.1.0–0.0.1.1	−10.777	0.000	0.000	*
7	0.0.0.0–0.1.0.0	−2.031	0.021	1.000	
8	0.0.0.1–0.1.0.0	6.176	0.000	0.000	*
9	0.0.1.0–0.1.0.0	3.970	0.000	0.004	*
10	0.0.1.1–0.1.0.0	9.999	0.000	0.000	*
11	0.0.0.0–0.1.0.1	−2.718	0.003	0.394	
12	0.0.0.1–0.1.0.1	−0.019	0.492	1.000	
13	0.0.1.0–0.1.0.1	−0.755	0.225	1.000	
14	0.0.1.1–0.1.0.1	1.591	0.056	1.000	
15	0.1.0.0–0.1.0.1	−1.981	0.024	1.000	
16	0.0.0.0–0.1.1.0	−3.070	0.001	0.128	
17	0.0.0.1–0.1.1.0	0.409	0.341	1.000	
18	0.0.1.0–0.1.1.0	−0.536	0.296	1.000	
19	0.0.1.1–0.1.1.0	2.455	0.007	0.846	
20	0.1.0.0–0.1.1.0	−2.078	0.019	1.000	
21	0.1.0.1–0.1.1.0	0.268	0.394	1.000	
22	0.0.0.0–0.1.1.1	−2.535	0.006	0.675	
23	0.0.0.1–0.1.1.1	−0.472	0.319	1.000	
24	0.0.1.0–0.1.1.1	−1.035	0.150	1.000	
25	0.0.1.1–0.1.1.1	0.771	0.220	1.000	
26	0.1.0.0–0.1.1.1	−1.988	0.023	1.000	
27	0.1.0.1–0.1.1.1	−0.365	0.358	1.000	
28	0.1.1.0–0.1.1.1	−0.618	0.268	1.000	
29	0.0.0.0–1.0.0.0	−13.562	0.000	0.000	*
30	0.0.0.1–1.0.0.0	4.972	0.000	0.000	*
31	0.0.1.0–1.0.0.0	0.693	0.244	1.000	
32	0.0.1.1–1.0.0.0	10.601	0.000	0.000	*
33	0.1.0.0–1.0.0.0	−3.509	0.000	0.027	*
34	0.1.0.1–1.0.0.0	0.869	0.192	1.000	
35	0.1.1.0–1.0.0.0	0.684	0.247	1.000	
36	0.1.1.1–1.0.0.0	1.122	0.131	1.000	
37	0.0.0.0–1.0.0.1	−6.887	0.000	0.000	*
38	0.0.0.1–1.0.0.1	−2.310	0.010	1.000	
39	0.0.1.0–1.0.0.1	−3.538	0.000	0.024	*
40	0.0.1.1–1.0.0.1	0.392	0.348	1.000	
41	0.1.0.0–1.0.0.1	−5.136	0.000	0.000	*
42	0.1.0.1–1.0.0.1	−1.183	0.118	1.000	
43	0.1.1.0–1.0.0.1	−1.757	0.039	1.000	
44	0.1.1.1–1.0.0.1	−0.539	0.295	1.000	
45	1.0.0.0–1.0.0.1	−3.699	0.000	0.013	*
46	0.0.0.0–1.0.1.0	−6.368	0.000	0.000	*
47	0.0.0.1–1.0.1.0	1.417	0.078	1.000	
48	0.0.1.0–1.0.1.0	−0.640	0.261	1.000	
49	0.0.1.1–1.0.1.0	5.456	0.000	0.000	*
50	0.1.0.0–1.0.1.0	−3.325	0.000	0.053	
51	0.1.0.1–1.0.1.0	0.501	0.308	1.000	
52	0.1.1.0–1.0.1.0	0.224	0.411	1.000	
53	0.1.1.1–1.0.1.0	0.835	0.202	1.000	
54	1.0.0.0–1.0.1.0	−0.944	0.173	1.000	
55	1.0.0.1–1.0.1.0	2.777	0.003	0.328	
56	0.0.0.0–1.0.1.1	−7.429	0.000	0.000	*
57	0.0.0.1–1.0.1.1	−3.192	0.001	0.085	
58	0.0.1.0–1.0.1.1	−4.329	0.000	0.001	*
59	0.0.1.1–1.0.1.1	−0.659	0.255	1.000	
60	0.1.0.0–1.0.1.1	−5.781	0.000	0.000	*
61	0.1.0.1–1.0.1.1	−1.735	0.041	1.000	
62	0.1.1.0–1.0.1.1	−2.386	0.009	1.000	
63	0.1.1.1–1.0.1.1	−1.003	0.158	1.000	
64	1.0.0.0–1.0.1.1	−4.475	0.000	0.000	*
65	1.0.0.1–1.0.1.1	−0.785	0.216	1.000	
66	1.0.1.0–1.0.1.1	−3.556	0.000	0.023	*
67	1.0.1.1–1.0.1.1	−3.411	0.000	0.039	*
68	0.0.0.0–1.1.0.0	2.869	0.002	1.000	
69	0.0.0.1–1.1.0.0	1.179	0.119	1.000	
70	0.0.1.0–1.1.0.0	6.216	0.000	0.000	*
71	0.0.1.1–1.1.0.0	−1.525	0.064	1.000	
72	0.1.0.0–1.1.0.0	1.172	0.121	1.000	
73	0.1.0.1–1.1.0.0	1.058	0.145	1.000	
74	0.1.1.0–1.1.0.0	1.362	0.087	1.000	
75	0.1.1.1–1.1.0.0	0.893	0.186	1.000	
76	1.0.0.0–1.1.0.0	3.632	0.000	0.017	*
77	1.0.0.1–1.1.0.0	1.365	0.086	1.000	
78	1.0.1.0–1.1.0.0	4.333	0.000	0.001	*
79	1.0.1.1–1.1.0.0	−5.654	0.000	0.000	*
80	0.0.0.0–1.1.0.1	−3.176	0.001	0.090	
81	0.0.0.1–1.1.0.1	−3.849	0.000	0.007	*
82	0.0.1.0–1.1.0.1	−1.668	0.048	1.000	
83	0.0.1.1–1.1.0.1	−4.873	0.000	0.000	*
84	0.1.0.0–1.1.0.1	−2.344	0.010	1.000	
85	0.1.0.1–1.1.0.1	−2.845	0.002	0.267	
86	0.1.1.0–1.1.0.1	−1.685	0.046	1.000	
87	0.1.1.1–1.1.0.1	−3.945	0.000	0.005	*
88	1.0.0.0–1.1.0.1	−1.686	0.046	1.000	
89	1.0.0.1–1.1.0.1	−3.490	0.000	0.029	*
90	1.0.1.0–1.1.0.1	−1.113	0.133	1.000	
91	1.0.1.1–1.1.0.1	−4.045	0.000	0.003	*
92	1.1.0.0–1.1.0.1	−4.196	0.000	0.002	*
93	0.0.0.0–1.1.1.0	−0.002	0.499	1.000	
94	0.0.0.1–1.1.1.0	−1.137	0.128	1.000	
95	0.0.1.0–1.1.1.0	2.439	0.007	0.884	
96	0.0.1.1–1.1.1.0	−2.875	0.002	0.242	
97	0.1.0.0–1.1.1.0	0.015	0.494	1.000	
98	0.1.0.1–1.1.1.0	−0.319	0.375	1.000	
99	0.1.1.0–1.1.1.0	0.424	0.336	1.000	
100	0.1.1.1–1.1.1.0	−1.307	0.096	1.000	
101	1.0.0.0–1.1.1.0	1.592	0.056	1.000	
102	1.0.0.1–1.1.1.0	−0.701	0.242	1.000	
103	1.0.1.0–1.1.1.0	2.288	0.011	1.000	
104	1.0.1.1–1.1.1.0	−1.630	0.052	1.000	
105	1.1.0.0–1.1.1.0	2.751	0.003	0.356	
106	1.1.0.1–1.1.1.0	−4.663	0.000	0.000	*
107	1.1.1.0–1.1.1.0	−2.450	0.007	0.857	
108	0.0.0.0–1.1.1.1	−3.052	0.001	0.136	
109	0.0.0.1–1.1.1.1	−1.107	0.134	1.000	
110	0.0.1.0–1.1.1.1	−4.009	0.000	0.004	*
111	0.0.1.1–1.1.1.1	−1.893	0.029	1.000	
112	0.1.0.0–1.1.1.1	−2.300	0.011	1.000	
113	0.1.0.1–1.1.1.1	−1.334	0.091	1.000	
114	0.1.1.0–1.1.1.1	−3.141	0.001	0.101	
115	0.1.1.1–1.1.1.1	−1.188	0.117	1.000	
116	1.0.0.0–1.1.1.1	−2.769	0.003	0.337	
117	1.0.0.1–1.1.1.1	−0.672	0.251	1.000	
118	1.0.1.0–1.1.1.1	−3.292	0.000	0.060	
119	1.0.1.1–1.1.1.1	0.294	0.384	1.000	
120	1.1.0.0–1.1.1.1	−2.176	0.015	1.000	

All *p*-values are adjusted using the Bonferroni correction method. The comparison labels (e.g., 0.0.0.1) represent the combinations of implementation strategies received by each group (1 = received, 0 = not received). The order of the implementation strategies received is calls, online meetings, arranged on-site meetings, and walk-in on-site meetings.

* Indicates *p* < .05.

#### Combination of strategies

Furthermore, groups that received the combination of arranged and walk-in on-site meetings reported higher activation numbers than other groups that received any other strategy or a combination of strategies (Table 3, e.g., comparison 5: 0.0.0.1–0.0.1.1, *Z*-value = −7.37, *p* < .001, *χ^2^* = 1,665.24).

The group receiving walk-in on-site meetings reported higher activation numbers compared to the group receiving arranged on-site meetings (Table 3, e.g., comparison 3: 0.0.0.1–0.0.1.0, *Z*-value = 4.88, *p* < .001, *χ^2^* = 1,665.24) and compared to the group receiving online meetings (Table 3, e.g., comparison 8: 0.0.0.1–0.1.0.0, *Z*-value = 6.18, *p* < .001, *χ^2^* = 1,665.24).

#### Correlation between strategies and activations

The relationship between the total number of employed strategies and the number of activations was analyzed. The correlation analysis showed a small to moderate positive linear relationship between the number of strategies employed and the number of activations (*r* = 0.30, *p* < .001). For each additional strategy employed, the associated number of activations increases by approximately 0.997. However, the model explains only approximately 8.87% of the variance in the number of activations. A simple linear regression was conducted to examine the predictive relationship between the total number of strategies and the number of activations. The regression model was statistically significant *F* (1, 24,815) = 2,415, *p* < .001.

## Discussion

This proof-of-concept study demonstrates the feasibility of applying the MOST framework to evaluate the effects of implementation strategies for increasing DiGA activations in real-world settings. A retrospective factorial design was utilized, enabling the systematic analysis of individual and combined implementation strategies. The identification of significant differences between groups, as well as the identification of combinations such as arranged and walk-in on-site meetings, illustrate the practical utility of the framework for optimizing implementation efforts. The results of this study underscore the potential of the MOST framework as a structured and efficient approach for addressing complex implementation challenges in digital health interventions.

As a methodological framework designed to optimize the development and evaluation of behavioral and psychosocial interventions, the MOST framework (28) is particularly useful for identifying the most effective combination of intervention components (here: implementation strategies).

In this study, we tested if the structure of the first two phases of the MOST framework can be applied when evaluating implementation strategies. For this purpose, we treated the implementation of digital health as the “intervention”, and specific “implementation strategies” as the “intervention components” according to MOST. We developed a logic model for DiGA implementation and conducted a retrospective factorial trial on the implementation strategies with the number of DiGA activations as the outcome.

The main goal of this study was to show the feasibility of a MOST trial using implementation strategies as components to inform future research projects, for example a full MOST trial including original data. We found that implementation strategies could be used as components in a MOST trial and that the conduct of a retrospective factorial experiment can lead to a deeper understanding of specific dependencies of implementation strategies or their combination for fostering the uptake of digital health applications.

As a secondary outcome, we used existing data to demonstrate what outcomes of a MOST trial to investigate implementation strategies would look like, and indicate the effect of implementation strategies such as calls, online-meetings, arranged and walk-in on-site-meetings on DiGA activation numbers. Groups of HCPs that received any implementation strategy demonstrated higher numbers of DiGA activations in comparison to groups that did not receive any implementation strategies. Notably, groups of HCPs receiving a combination of arranged and walk-in on-site meetings showed higher activation numbers compared to those receiving other strategies. This indicates that a multi-faceted approach with face-to-face engagement, whether scheduled or walk-in, may be beneficial in increasing the number of DiGA activations. These findings are consistent with those of previous studies, including a systematic review indicating that multi-faceted implementation strategies are more effective than uni-faceted strategies (24).

The moderate positive correlation between the total number of strategies employed and the total number of activations suggests that while employing more strategies can lead to increased activations, this relationship alone has limited explanatory power. Contrary to our findings, the literature points in a different direction. A systematic review examining the effectiveness of implementation strategies among HCPs in a cancer care context found no significant association between the number of strategies employed and behavioral changes among HCPs (37). This result might be due to limitations in our study setup. Addressing the behavioral change of HCPs might require a more targeted approach.

### Limitations to using existing data for a retrospective factorial design

Although this study effectively employed the MOST framework to assess implementation strategies, several constraints affected the framework's comprehensive utility and effectiveness, particularly with regard to data structure and methodological limitations. A significant limitation of this proof-of-concept study is the potential feedback loop between the dependent variable (DiGA activations) and the independent variable (implementation strategies). The differing approaches employed on HCPs who already prescribe more DiGA vs. those who do not, may influence the implementation strategies used. This creates a bias, as HCPs inclined to prescribe DiGA might receive different and/or more intensive strategies, potentially skewing the results. Thus, it is challenging to determine if the increase in DiGA activations is due to the strategies or pre-existing tendencies of the HCPs.

HelloBetter documents various implementation strategies, including calls, emails, faxes, meetings, postal mails, letters, test accounts, flyers, webinars, congresses, and campaigns. For this study, we focused specifically on in-person implementation strategies such as calls, online meetings, arranged and walk-in on-site meetings. This limited scope might not fully represent the broader implementation efforts employed by HelloBetter.

Additionally, we did not focus on a specific time frame while evaluating implementation strategies, which is crucial as the employed strategies underwent modifications over the course of the data collection period. This results in difficulties in attributing changes in DiGA activation numbers to specific strategies or time periods, as the evolving nature of the strategies introduces variability and potential confounding factors in the results. The retrospective factorial design also presents limitations, including the inability to control for confounding variables or establish causality. Participants were not randomly assigned to the groups, which may introduce biases. Additionally, the order of implementation activities was not counterbalanced, which might potentially lead to order effects. Furthermore, the non-parametric and underpowered analyses compromise the robustness and generalizability of the findings.

The presence of such biases introduces complications into the optimization process within the MOST framework, as it becomes challenging to isolate the effects of the implementation strategies from the influence of the pre-existing behaviors of the HCPs. The presence of feedback loops, the absence of a defined time frame and the absence of randomization make it challenging to establish clear causal relationships between the implementation strategies and the observed outcomes. This, in turn, constrains the MOST framework's capacity to accurately assess the effectiveness of the strategies and further constrains the framework's capacity to conduct a comprehensive and reliable optimization process.

For this study, we had no access to prescription numbers of DiGA, only to the activation numbers. This means that there might be a number of prescriptions issued by the HCPs that did not turn into an activation by the patient. Therefore, HCPs might have prescribed more DiGA than we assess in this study. Still, there are advantages to focusing on activation numbers rather than prescription numbers. The process allows for the evaluation of the practical adoption of DiGA by end-users. While prescriptions depict direct HCP behavior and are an important intermediate metric, they do not necessarily reflect whether patients engage with and activate the prescribed DiGA, which should be the ultimate health benefit outcome. Previous research has shown that a substantial proportion of prescribed DiGA are never activated, highlighting a key gap between prescription and actual utilization [e.g., (19)]. For example, a HCP might issue a high number of DiGA prescriptions, but to an unfit group of patients, which would in turn not activate their DiGA. Or, a HCP might be unable to explain the benefit of the DiGA prescription to the patient, resulting in a non-activation. Such prescriptions would then also not depict implementation success. Therefore, activation numbers provide a more direct measure of DiGA uptake and use, which are critical indicators of the intervention's success in real-world settings. Future analyses could benefit from examining both prescription and activation numbers as well as why not all prescriptions are turned into DiGA activations to gain a more comprehensive understanding of HCP and DiGA user behavior. In the context of the MOST framework, the distinction between prescriptions and activations is of critical importance, as the framework is designed to optimize and evaluate the most effective components of an intervention. The consideration of both metrics would facilitate a more nuanced analysis of the influence of implementation strategies on both HCP behavior (prescriptions) and end-user engagement (activations).

Despite its limitations, this study offers valuable insights into digital mental health implementation science. The findings of our study indicate that the MOST framework might be an appropriate tool for the assessment of implementation strategies. However, the results also highlight the necessity of integrating frameworks such as MOST into the planning and optimization phase of strategies before implementing them.

### Practical implications and recommendations

The findings of this study offer several actionable insights for researchers and implementers working on the investigation and improvement of the implementation of digital interventions. This study implies a method to critically investigate implementation activities in routine care settings. The outcome of such research would be quantifiable knowledge on the effects of specific implementation strategies. Specifically digital healthcare providers, such as DiGA providers, could critically investigate and then adapt their implementation strategies based on tangible measures. Implementation can be costly, failed implementation even more so. It has been reported that the uptake of health care innovations into routine care takes significant time and effort (38). Speeding up the process of integrating a health care innovation, such as DiGA, into routine care might be crucial to its success and sustainability. We assume that too often, implementers use an “it seems like a good idea” approach for choosing and applying implementation strategies, without strategically evaluating their success. Applying the MOST framework to assess the effect of specific implementation strategies might improve the effectiveness of implementation endeavors, such as integrating DiGA into German healthcare. To leverage these insights, healthcare systems should prioritize integrating the evaluation and optimization of implementation strategies into routine workflows, ensuring sufficient training and resources for implementation staff. Policymakers can support this by providing incentives for HCPs to participate in such strategies, such as certification for training.

While this proof-of-concept study gives an indication that it is possible to conduct a MOST trial to systematically assess the effects of specific implementation strategies using existing data, future research should run a full MOST trial with original data to assess the effect of implementation strategies. Within the preparation phase, researchers should evaluate all ongoing and planned implementation activities as well as their assumed effects. In the best case scenario, this phase would start before the actual implementation of an innovation, so that all implementation activities could be purposefully selected. If implementation activities are already ongoing, researchers could use specific techniques such as stakeholder involvement to find consensus on the most promising implementation strategies for their context. Either way, this process should include a full implementation evaluation, for example using the CFIR framework (26), to evaluate the context as well as barriers and facilitators of the implementation. Then, implementation strategies should be tailored to these findings.

After identifying implementation strategies, components of such to test (for example the digital vs. face-to-face delivery of the strategy) or multi-faceted sets of strategies, researchers would conduct the factorial experiment. They could also choose alternative approaches to investigate strategy effectiveness, such as fractional factorial experiments, sequential, multiple assignment, randomized trials (SMARTs), micro-randomized trials, system identification, or other (28). Researchers would collect data in the specific context, randomly assigning HCPs to a group receiving a defined set of implementation strategies (or none). Running this factorial implementation experiment would allow for the investigation of effects of implementation strategies and their combinations. By conducting this trial, most of the above mentioned limitations could be overcome. The trial could be powered accordingly and groups for the factorial would be set. To refer to our example, groups of HCPs would be randomized to the conditions of the factorial design and these HCPs would then only receive these specific numbers of strategies. This procedure would then allow for a less biased assessment of specific strategies.

After this optimization phase, in a full MOST trial the researcher proceeds to the evaluation phase of MOST. This phase involves testing effectiveness through a RCT to determine whether the optimized implementation produces show a statistically and clinically significant effect, measured as the difference between the optimized implementation intervention and control groups (e.g., “implementation-as-usual”). If the RCT confirms the implementation intervention's effectiveness, it can be prepared for roll-out in the intended setting.

Limitations of this procedure include the limitation of the number of components, confounding factors for such a trial and the high costs of such a trial. Naturally, a high number of implementation activities are carried out at once when implementing digital interventions. Researchers investigating the effects of specific implementation strategies would need to carefully consider which activities constitute as implementation strategies to be investigated and how many components they would want to include in their factorial design. Likewise, they would have to be aware of the confounding effects of all implementation strategies going at the implementation site, not being considered implementation activities within the factorial design. Lastly, we assume that such a MOST trial for the assessment of the effectiveness of implementation strategies for digital interventions will be quite costly. There will be direct costs because of big sample sizes due to the low expected effect size for separate implementation strategies. Indirect costs might include lower activation numbers in those conditions of the factorial design with less effective strategies and groups receiving no strategies for the time of the trial. Naturally, implementers might use the “the more the better” method, applying multiple implementation strategies at once and seeing an effect of those strategies. This might lead to short-term success of this “blanket approach”, but might be more costly in the long-term than investing in the investigation of the most successful implementation strategies. Nonetheless, the risk of conducting a factorial design and applying (a group of) less effective implementation strategies for a group of HCPs might make implementers and companies hesitant to invest in such a trial.

Lastly, implementers would have to decide if they wanted to conduct the third phase of the MOST framework and would evaluate the optimized intervention through a randomized controlled trial (RCT) or another rigorous study design. By doing this, implementers could confirm the efficacy and effectiveness of the implementation intervention (the single or combination of implementation strategies) as a whole, ensuring that the chosen configuration of strategies (components) is effective in practice. Such investigations into implementation strategies have been very rare and would also foster the field of implementation science.

For the example of DiGA implementation, running a full MOST trial would enable DiGA implementers and providers to understand the working mechanisms of DiGA implementation further. For now, much focus has been placed on the identification and discussion of implementation barriers. Overcoming those barriers has mainly resulted in classical marketing and sales activities. Utilizing the knowledge of the field of implementation science and then systematically investigating the effectiveness of specific implementation strategies might overcome barriers to DiGA implementation and foster the uptake of those interventions in routine care.

## Conclusion

In conclusion, this proof-of-concept study demonstrates the feasibility of applying the MOST framework to evaluate effects implementation strategies for increasing DiGA activations in real-world settings. By employing the preparation and optimization phases of the MOST framework, this study highlights its potential as a structured approach for systematically evaluating and optimizing implementation efforts. The application of the framework enabled the systematic comparison of multiple strategies, providing actionable insights into their relative effectiveness.

While this study focuses on the feasibility of using the MOST framework rather than delivering definitive conclusions on implementation strategy effectiveness, the findings suggest that the framework can serve as a valuable tool for digital health providers and policymakers. Future research should build upon this work by conducting fully powered trials to further validate the framework's applicability and optimize implementation strategies, ultimately supporting broader adoption of digital health interventions and improving access to mental health care for a broader population.

## Data Availability

The datasets presented in this article are not readily available because they belong to the company HelloBetter. Access to the data requires a formal request to be made directly to HelloBetter. Requests to access the datasets should be directed to kontakt@hellobetter.de.
